# Complicated Jejunal Diverticulosis as a Rare Cause of Small Bowel Perforation in an Immunocompromised Patient: A Case Report

**DOI:** 10.7759/cureus.110893

**Published:** 2026-06-15

**Authors:** Andrea Navalón Calzada, Oscar Gerardo Chavéz Cadena, Paola Gabriela Fernández Pérez, Oscar Arturo Amaro Vázquez, Carlos Mancera Steiner

**Affiliations:** 1 Department of General Surgery, Hospital Español, Mexico City, MEX; 2 Department of Oncologic Surgery, Hospital Español, Mexico City, MEX

**Keywords:** diverticulitis, immunocompromised host, intestinal perforation, jejunal diverticulitis, pneumatosis intestinalis, small bowel diverticulosis

## Abstract

Jejunal diverticulosis is an uncommon and often underrecognized condition that may lead to life-threatening complications, particularly in immunocompromised patients. Among these, perforation remains an uncommon but critical event, with variable clinical presentations depending on the degree of peritoneal contamination. We present the case of a 74-year-old male with a history of renal transplantation on chronic immunosuppressive therapy who presented with acute abdominal pain, fever, and diarrhea. Laboratory findings were consistent with systemic inflammation and poor nutritional status. Contrast-enhanced abdominal CT demonstrated a saccular outpouching in the proximal jejunum, mesenteric pneumatosis, and contained extraluminal contrast leakage, suggestive of complicated jejunal diverticulitis. Exploratory laparotomy revealed a jejunal perforation directed toward the mesenteric border, with intestinal content leakage confined to the mesentery and no evidence of free intraperitoneal contamination. Segmental resection with primary anastomosis was performed, resulting in a favorable postoperative course. This case highlights the importance of recognizing contained jejunal perforation as a distinct clinical entity, particularly in immunocompromised patients, in whom early diagnosis and timely surgical management are essential to prevent progression and improve outcomes.

## Introduction

Jejunal diverticulosis is a rare clinical entity, with an incidence ranging between 0.3% and 2.3% in the general population [1,2]. While the majority of cases remain asymptomatic, this condition can lead to severe and potentially life-threatening complications, including diverticulitis, hemorrhage, intestinal obstruction, and perforation [2,3]. Due to its nonspecific clinical presentation, jejunal diverticulitis is frequently misdiagnosed as more common acute abdominal conditions, such as appendicitis, peptic ulcer disease, or Crohn’s disease [3]. Although perforation is an uncommon complication, occurring in only 2-6% of symptomatic cases, it carries an exceptionally high mortality rate that can exceed 20-40% if surgical intervention is delayed [1,4]. This diagnostic challenge is further amplified in immunocompromised individuals, in whom the classic signs of peritoneal irritation and systemic inflammation may be blunted or delayed by chronic immunosuppressive therapy. While free purulent peritonitis is the most frequent consequence of transmural rupture, a contained mesenteric perforation represents an even rarer clinical scenario that mimics other causes of acute abdomen [5]. The novelty of the present case lies in the description of this unusual contained mesenteric perforation in a renal transplant recipient under chronic maintenance immunosuppression. This scenario demands an exhaustive initial differential diagnosis and a precise understanding of how targeted therapies can mask catastrophic intra-abdominal progression, underscoring the vital importance of early surgical recognition to optimize clinical outcomes.

## Case presentation

A 74-year-old male patient with a medical history of systemic arterial hypertension and IgA nephropathy diagnosed in 1995, with progression to chronic kidney disease requiring renal transplantation in 2016, was currently on chronic long-term (10-year) immunosuppressive therapy with prednisone, tacrolimus, and mycophenolic acid. Additional relevant history includes right femoral deep vein thrombosis and pulmonary embolism in 2023, for which he is on oral anticoagulation.

The current illness began one day prior to admission to the ED with the sudden onset of oppressive abdominal pain located in the upper abdomen, rated 10/10 in intensity, and radiating to the hypogastrium, left iliac fossa, and left flank. The patient also reported a fever up to 37.7 °C, chills, and three loose stools classified as Bristol type 6. Due to the persistence and worsening of these symptoms, he sought medical attention.

On physical examination, the patient was conscious and oriented, with vital signs within normal parameters. The abdomen was globose due to a prominent adipose panniculus but remained soft and depressible, with hypoactive peristalsis and metallic sounds in the epigastrium. There was tenderness on superficial, medium, and deep palpation along the colonic frame and right iliac fossa, tympanism on percussion, and generalized rebound tenderness, which was more pronounced in the left abdomen. Given the patient’s complex background, specifically chronic immunosuppression and oral anticoagulation, the initial differential diagnosis in the ED was broad. It included acute mesenteric ischemia (highly suspected due to his thromboembolic history), atypical perforated peptic ulcer disease, acute severe pancreatitis, and mechanical bowel obstruction. Notably, his baseline immunosuppressive regimen was suspected of blunting the expected classic rigid signs of generalized peritonitis, presenting instead with a softer abdomen despite the underlying severe pathology. Trough serum tacrolimus levels were not drawn during the acute evaluation due to the emergency nature of the presentation and the immediate requirement for advanced diagnostic imaging.

Laboratory evaluation on admission was remarkable for significant leukocytosis with a left shift, acute kidney injury, and markedly elevated inflammatory markers. Additionally, mild-to-moderate anemia and signs of malnutrition, including hypoproteinemia and hypoalbuminemia, were documented. Urinalysis was positive for both microhematuria and pyuria. The complete laboratory profile on admission is detailed in Table 1.

**Table 1:** Baseline laboratory findings on admission

Laboratory parameter	Patient value	Reference range (adults)
Complete blood count		
Leukocytes (WBC)	20 × 10⁹/L	4.5-11.0 × 10⁹/L
Neutrophils	84.9%	40-75%
Bands	5%	0-5%
Hemoglobin	9.3 g/dL	13.5-17.5 g/dL
Hematocrit	29.4%	41-50%
Inflammatory markers		
C-reactive protein	20.23 mg/L	<5.0 mg/L
Erythrocyte sedimentation rate	56 mm/h	<15 mm/h
Procalcitonin	1.23 ng/mL	<0.15 ng/mL
Basic metabolic panel		
Total proteins	5.7 g/dL	6.0-8.3 g/dL
Albumin	2.98 g/dL	3.5-5.0 g/dL
Serum creatinine	1.75 mg/dL	0.7-1.3 mg/dL
Urinalysis		
Erythrocytes	25 erythrocytes/µL	Negative (<10 erythrocytes/µL)
Leukocytes	50 leukocytes/µL	Negative (<10 leukocytes/µL)

Simple abdominal CT with oral contrast revealed an inflamed diverticulum located approximately 30 cm from the ligament of Treitz. Additionally, at 40 cm, a jejunal perforation was observed on the mesenteric border, with extravasation of intestinal contents strictly confined to the mesentery, consistent with a contained perforation. No evidence of free intraperitoneal contamination or pneumoperitoneum was found (Figures 1-3).

**Figure 1:**
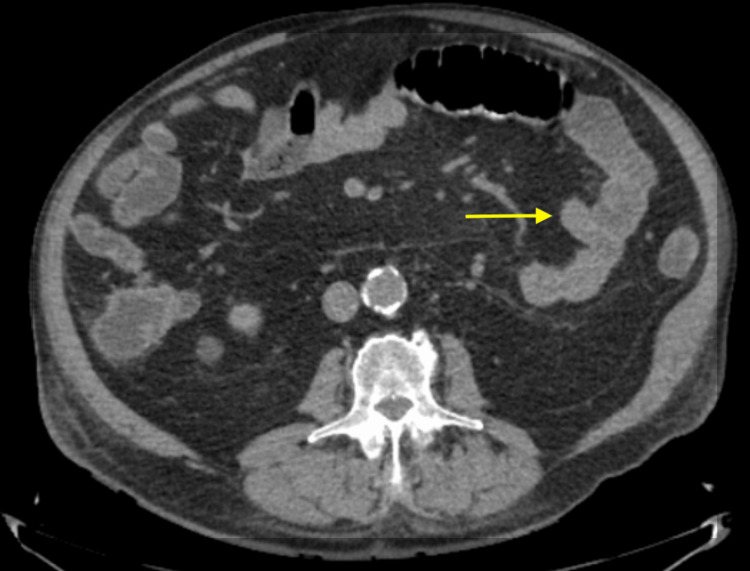
Axial abdominal CT scan Saccular formation on the mesenteric border of the proximal jejunum (arrow).

**Figure 2:**
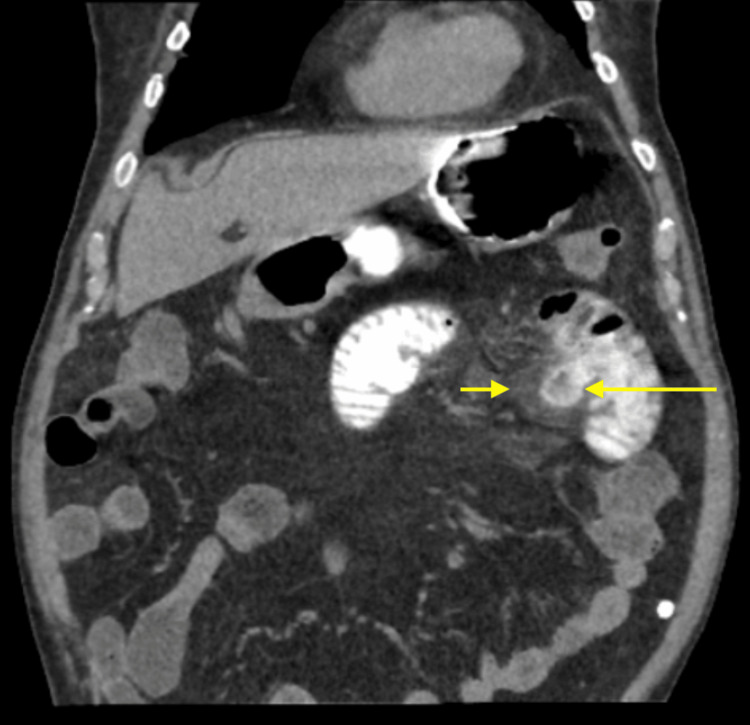
Coronal abdominal CT scan with oral contrast Dilatation of the proximal jejunum up to 42 mm, with a saccular formation on the mesenteric border (large arrow), distal caliber reduction, and wall thickening, associated with surrounding fat stranding (small arrow).

**Figure 3:**
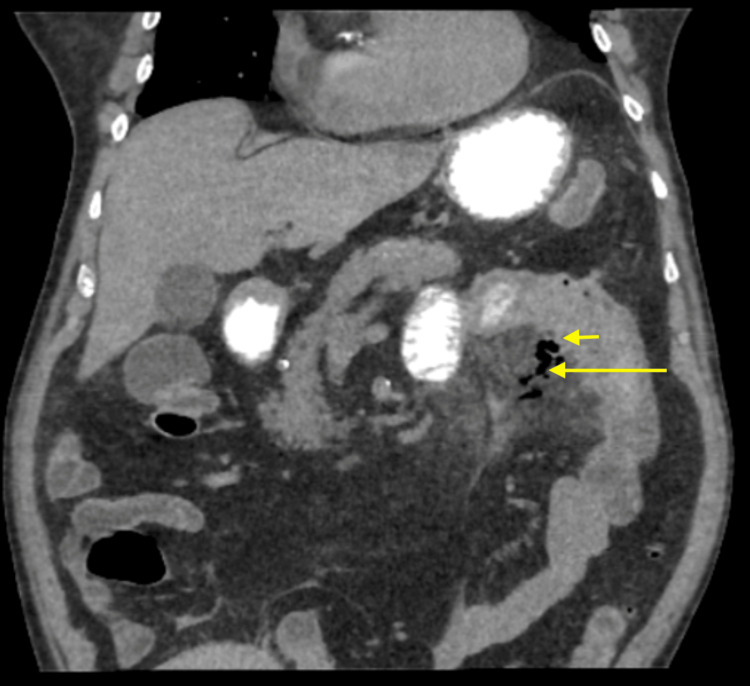
Coronal abdominal CT scan with oral contrast Extraluminal gas (large arrow) and a subtle leak of oral contrast adjacent to the mesenteric border of the jejunum (small arrow).

Based on these findings, the patient was admitted to the intermediate care unit with a diagnosis of complicated jejunal diverticulitis with a contained intestinal perforation, likely secondary to chronic corticosteroid and immunosuppressive therapy. Given the patient’s critical combination of chronic immunosuppression and severe protein-energy malnutrition, evidenced by a Nutritional Risk Screening 2002 score ≥ 5, severe hypoalbuminemia (2.98 g/dL), and hypoproteinemia (5.7 g/dL), immediate emergency surgery carried an unacceptably high risk of anastomotic leak. Therefore, a planned 48-hour window for optimization was targeted, which allowed for the strict correction of fluid-electrolyte imbalances. During this time, he was kept under close clinical surveillance, oral anticoagulation was temporarily suspended, and nutritional status was aggressively optimized via total parenteral nutrition (TPN) administered through a central venous catheter, formulated to provide 25 kcal/kg/day with a high amino acid composition (1.5 g/kg/day) to counteract the catabolic state and minimize postoperative anastomotic complications.

Due to the high risk of progression in an immunocompromised host, an elective exploratory laparotomy was performed 48 hours after admission. Surgical exploration identified a fixed loop 30 cm from the ligament of Treitz with an inflamed, non-perforated diverticulum. At 40 cm, a jejunal perforation was encountered with extensive fibrin deposits and localized intestinal leakage confined to the mesentery (Figure 4); fluid cultures were obtained. A segmental small bowel resection was performed, leaving a proximal margin 25 cm from the fixed loop and a distal margin 10 cm from the perforation using a GIA stapler; both stapled stumps were reinforced with interrupted Lembert sutures. A second, incidental diverticulum was identified in the proximal stump and was included in the resected segment; the remainder of the jejunum was macroscopically intact. A 40-cm specimen was successfully removed and sent for definitive histopathological analysis. Intestinal continuity was restored via a side-to-side, antimesenteric stapled anastomosis, followed by closure of the mesenteric defect with a continuous suture. After thorough abdominal lavage and aspiration, the procedure concluded without incident, and the patient was transferred to the recovery room hemodynamically stable.

**Figure 4:**
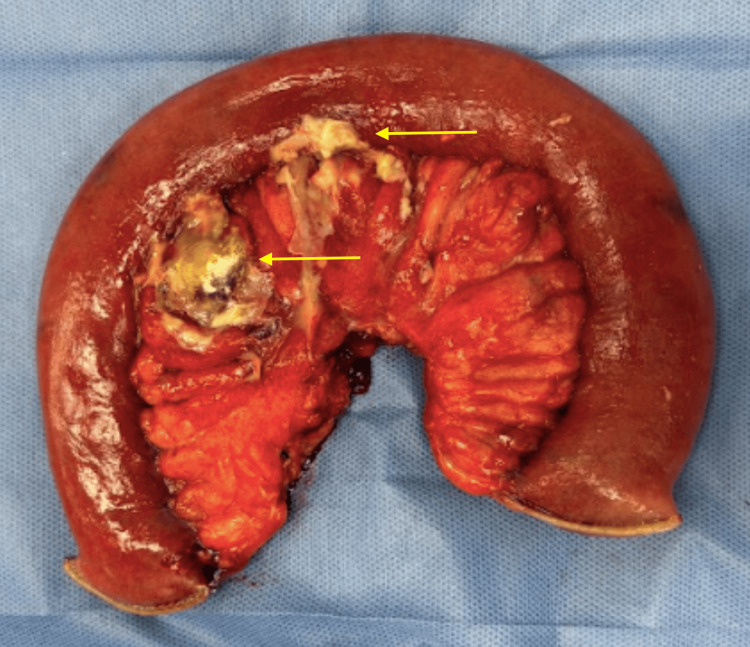
Gross specimen of the resected jejunal segments Jejunal mesenteric border perforation with fibrinous deposits and intestinal leakage.

The postoperative course was favorable, with the patient remaining afebrile and hemodynamically stable. Empirical IV antibiotic therapy was administered with ertapenem (1 g q24h) and vancomycin (1 g q24h). Immunosuppressive therapy was carefully resumed on postoperative day (POD) 3, using IV hydrocortisone (100 mg q8h) and oral tacrolimus (4 mg q24h).

The patient remained nil per os (NPO) with TPN support during the first week. A follow-up abdominal CT on POD 7 confirmed the integrity of the anastomosis, allowing the initiation of oral liquids, which were progressively advanced to a soft diet without complications. The patient was discharged home on POD 10 in good general condition, tolerating oral intake and with adequate pain control.

Histopathological examination subsequently confirmed the diagnosis of complicated jejunal diverticulosis with acute perforated diverticulitis and perimesenteric abscess formation. Gross examination of the resected jejunal segment (Figure 5) clearly demonstrated multiple wide-mouthed diverticula projecting from the mesenteric border, with a visible transmural perforation leading into an area of necrotic and inflamed mesenteric tissue, consistent with the contained abscess seen on imaging. Microscopic evaluation subsequently corroborated these findings, revealing acute fibrinopurulent serositis, extensive mesenteritis, mild chronic ileitis, and focal areas of recent mural hemorrhage within the diverticular wall.

**Figure 5:**
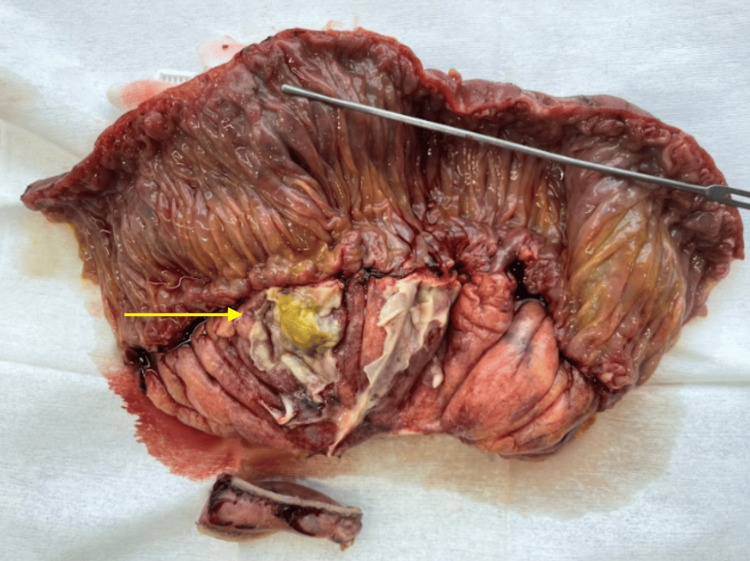
Gross specimen of the resected jejunal segment Saccular mucosal invagination toward the mesentery, surrounded by friable, greenish-gray material.

## Discussion

Jejunal diverticula are more frequently observed than ileal diverticula, a distribution attributed to the larger caliber of penetrating jejunal arteries [3]. Jejunal diverticulosis occurs predominantly in elderly patients, with a mean age of presentation in the seventh decade of life and a male predominance [2,5], matching the demographic profile of the patient presented here. These lesions are typically multiple and characteristically located along the mesenteric border of the jejunum, unlike Meckel’s diverticulum, which is solitary and antimesenteric [1,3]. Complications arise in approximately 10-20% of cases, with acute diverticulitis being the most common clinical manifestation [3,6].

Most jejunal diverticula are pseudodiverticula, consisting of mucosal and submucosal herniation through areas of muscular weakness where the vasa recta penetrate the muscularis propria [1]. Abnormal peristalsis, intestinal dyskinesia, and elevated intraluminal pressures are considered the primary etiologic factors [1]. This localized stasis predisposes to bacterial overgrowth, inflammation, microperforation, and eventual macroperforation [2]. In our patient, chronic immunosuppressive therapy, specifically corticosteroids, likely played a synergistic role by causing mucosal thinning and undermining structural tissue integrity, thereby accelerating the progression from localized inflammation to a contained perforation.

Symptoms of jejunal diverticulitis are notoriously nonspecific and include abdominal pain, bloating, nausea, vomiting, and altered bowel habits [3,6]. When complicated, patients may present with frank peritonitis, gastrointestinal bleeding, or small bowel obstruction [2,3]. Due to the symptomatic overlap with other acute abdominal conditions (such as acute appendicitis, cholecystitis, or colonic diverticulitis), definitive diagnosis is often significantly delayed [4,7]. This clinical challenge is further heightened in immunocompromised hosts, in whom chronic steroid and immunosuppressant use can blunt the host’s inflammatory response, masking classic signs of peritonitis and leading to atypical clinical presentations, such as the self-limiting diarrhea and absence of initial severe tachycardia observed in this case.

CT remains the gold standard for identifying jejunal diverticulitis and its complications, demonstrating round, extraluminal structures containing air or fluid, localized wall thickening, and mesenteric fat stranding [1,8]. While CT is highly sensitive for detecting perforation, abscesses, or pneumoperitoneum [3], advanced local inflammation can sometimes obscure the anatomy, mimicking a neoplastic process or requiring exploratory laparotomy for definitive confirmation [3,7]. In this case, the CT scan was highly precise, accurately identifying both the inflamed outpouching and the contained nature of the perforation, which allowed for safe intermediate-care monitoring and optimized preoperative nutritional planning. However, in the initial emergency setting, the rational use of basic imaging resources, such as plain abdominal and upright chest X-rays, remains an indispensable first step. These accessible studies are crucial for the rapid demonstration of pneumoperitoneum or mechanical obstruction, enabling clinicians to make accelerated surgical decisions, avoid unnecessary institutional expenses, and prevent catastrophic delays in patients with frank hollow viscus perforation [9].

Currently, there are no standardized clinical guidelines for the management of jejunal diverticulitis; hence, treatment must be tailored to the patient’s clinical stability and radiologic findings [3,7]. Nonoperative management consisting of IV fluids, strict bowel rest (NPO), and broad-spectrum antibiotics is appropriate for localized, uncomplicated disease without evidence of perforation [1]. In selected stable cases, percutaneous drainage may be employed for contained abscesses [3]. Conversely, immediate surgical intervention is mandated in the presence of generalized peritonitis, uncontrolled sepsis, free pneumoperitoneum, or ongoing hemorrhage [1,4].

In our patient, although conservative management with broad-spectrum antibiotics was initially successful in maintaining clinical stability due to the contained nature of the perforation, definitive surgical resection was selected rather than long-term conservative treatment. In a renal transplant recipient under triple immunosuppressive therapy, the capacity for tissue healing is significantly impaired. The literature demonstrates that immunocompromised individuals carry an exceptionally high risk of secondary conservative management failure, recurrent diverticulitis, or unpredictable delayed free perforation into the peritoneal cavity [9,10]. Therefore, our case demonstrates that a middle-ground approach, such as initial intermediate care monitoring, aggressive nutritional optimization via targeted TPN [11], and temporary NPO status followed by timely, scheduled surgical intervention, is a highly viable and safe strategy for contained mesenteric perforations within the high-risk context of posttransplant immunosuppression. The surgical procedure of choice remains segmental resection with primary anastomosis, which should be restricted to the affected segment to minimize the risk of short bowel syndrome [3,7]. Reported negative prognostic factors include advanced age, significant comorbidities, and delayed diagnosis [3], making early multidisciplinary optimization key to achieving the favorable outcome seen in our patient.

## Conclusions

This case highlights the clinical importance of considering complicated jejunal diverticulitis with contained perforation as a differential diagnosis in immunocompromised patients presenting with an atypical acute abdomen. Managing these high-risk surgical candidates requires a nuanced and highly individualized approach. Our experience suggests that when imaging findings confirm that a perforation is strictly contained and the patient remains hemodynamically stable without systemic sepsis, a brief, carefully monitored period of intensive optimization in an intermediate care setting may reduce the risk of anastomotic complications. However, because these findings are based on a single clinical scenario, this strategy cannot be universally generalized, and immediate surgical intervention must remain the standard of care if clinical deterioration occurs. Ultimately, tailored multidisciplinary optimization can lead to successful surgical resection and favorable outcomes in highly selected cases.
